# Four Mitochondrial Genomes of Buprestinae (Coleoptera: Buprestidae) and Phylogenetic Analyses

**DOI:** 10.3390/genes16070828

**Published:** 2025-07-16

**Authors:** Yingying Li, Jieqiong Wang, Bowen Ouyang, Zhonghua Wei, Aimin Shi

**Affiliations:** 1 College of Life Sciences, China West Normal University, Nanchong 637009, China; yingyingli045@163.com (Y.L.); jqwang0902@126.com (J.W.); oybw2001@163.com (B.O.); aiminshi@cwnu.edu.cn (A.S.); 2 The Key Laboratory of Southwest China Wildlife Resources Conservation of the Ministry of Education, College of Life Sciences, China West Normal University, Nanchong 637009, China

**Keywords:** jewel beetles, *Buprestis*, *Chrysobothris*, *Phaenops*, phylogenetics

## Abstract

Background: The family Buprestidae is one of the largest families in Coleoptera; however, the number of reported mitochondrial genomes for this family is limited. Methods: In this study, mitogenomes of *Chrysobothris violacea*, *C. shirakii*, *Buprestis fairmairei,* and *Phaenops yin* were sequenced, assembled, and annotated. The mitogenomes of *Chrysobothris*, *Phaenops,* and *Buprestis* are reported for the first time. Results: The mitogenomes of *Chrysobothris violacea*, *C. shirakii*, and *Phaenops yin* are complete, while the mitogenome of *Buprestis fairmairei* is partial, lacking trnV and 12S genes. The AT-skew of these four mitogenomes is positive (0.02–0.09). Among the protein-coding genes, the Ka/Ks ratio for cox1 is the lowest (0.05), and the nucleotide diversity for nd6 is the highest. Conclusions: The phylogenetic trees based on mitogenome sequences suggest that the target genus *Chrysobothris* is sister to *Phaenops*, and the target genus *Buprestis* is sister to (*Melanophila* + (*Chrysobothris* + *Phaenops*)) clade. The results of this study will provide mitogenomic data for further research on the mitogenome and phylogeny of Buprestidae.

## 1. Introduction

The Buprestidae is one of the largest families in Coleoptera, comprising six subfamilies and more than 15,000 species worldwide [1]. All the adult forms of Buprestidae are phytophagous insects, except *Xyroscelis crocata* (Gory & Laporte, 1839) feeding on the sap of *Macrozamia communis* [2]. Some species, such as *Agrilus planipennis* (Fairmaire, 1888), *Agrilus mali* (Matsumura, 1924), *Lamprodila festiva* (Linnaeus, 1767), and *Melanophila acuminata* (DeGeer, 1774), are known as forestry pests or invasive species [3,4,5,6,7]. Buprestid beetles are very widely distributed and can be found in almost all terrestrial and insular habitats where green plants exist. Generally, species with wood-boring habits are primarily distributed in temperate and subtropical regions, while those with leaf-mining habits are more common in moist tropical regions [8].

Although the classification system proposed by Bellamy [1] is widely used, high-level phylogenetic relationships from that system have come into question, including the monophyly of Buprestinae and Chrysochroinae [9]. The mitochondrial genome is an important molecular marker in animals. With the rapid development of sequencing technologies, the extraction, sequencing, and assembly of mitogenome have become much easier. To date, mitogenomes have been widely used for phylogenetic studies of insects [10,11,12,13]. The insect mitogenome, a double-stranded and closed molecule, contains 13 protein-coding genes (PCGs), 22 transfer RNA genes (tRNAs), two ribosomal RNA genes (rRNAs), and a control region (CR) also called A + T-rich region [14]. The length of the complete mitogenome of Buprestidae ranges from 15 to 16 kbp [15]. Although 31 mitogenomes have been sequenced and annotated in Buprestidae, those of Buprestinae remain limited.

In the present study, the mitogenomes of *Chrysobothris violacea* (Kerremans, 1892), *Chrysobothris shirakii* (Miwa & Chûjô, 1935), *Buprestis fairmairei* (Théry, 1910), and *Phaenops yin* (Kubáň & Bílý, 2009) were sequenced, assembled, and annotated. The base composition, gene rearrangement, relative synonymous codon usage (RSCU), nucleotide diversity (Pi), and ratio of non-synonymous substitutions (Ka) to synonymous substitutions (Ks) were analyzed and compared. The phylogenetic relationships of subfamilies in Buprestidae were studied based on the mitogenomic data, including four new mitogenome sequences provided in this study.

## 2. Materials and Methods

### 2.1. Sampling and DNA Extraction

The specimen of *Chrysobothris violacea* was collected from Xinhua Village, Weideng Township, Weixi County, Yunnan Province, China on 1 August 2023. The specimens of *Chrysobothris shirakii* were collected from Yangjiayu, Yuguan Township, Qinhuangdao City, Hebei Province, China on 6 May 2024. The specimens of *Buprestis fairmairei* were collected from Baijixun Township, Weixi County, Yunnan Province, China on 29 Jun 2021. The specimens of *Phaenops yin* were collected from Dapingshan, Mianning City, Sichuan Province, China on 22 July 2023. The adults of *Phaenops yin* were discovered on the wood of burn areas, while some larvae were found under the bark. Specimens of adult form were stored in 95% alcohol and then stored at −20 °C when returned to the laboratory. All these specimens were identified based on the original descriptions [16,17,18,19]. In order to ensure sequencing quality, specimens were sequenced as soon as possible after collection and identification. All the studied specimens were deposited in Museum of China West Normal University. The tissues of thorax and legs were used for total DNA extraction, following the protocol of the Ezup Column Animal Genomic DNA Purification Kit (Shanghai, China). The Illumina libraries were constructed with an average insert size of 350 bp and then sequenced using Illumina platform with a short paired-end strategy (150 bp).

### 2.2. Mitogenome Assembly, Annotation, and Analysis

To obtain the target reads, Trimmomatic v. 0.36 [20] was used to remove low-quality bases and adapter sequences from the raw data. Subsequently, IDBA-UD v. 1.1.1 [21] was employed to assemble the target reads. The identification of open reading frames led to the discovery of 13 PCG genes. Additionally, the analysis of tRNA gene boundaries facilitated the recognition of two rRNA genes, as well as the A + T-rich region. Finally, the contigs were assembly using Geneious v. 11.0.2 [22]. The mitogenomic maps were drawn using Organellar Genome DRAW [23].

The mitogenomic annotation was conducted using online tool Mitos WebServer [24]. The predicted secondary structures of 22 tRNA genes were visualized using VARNA v. 3.9 [25] based on the result of annotation completed by Mitos. The base composition of mitogenome sequences and the relative synonymous codon usage (RSCU) of 13 PCG genes were calculated and analyzed using MEGA v. 10.0.2 [26]. The AT-skew and GC-skew of four mitogenome sequences were calculated based on the formula proposed by Perna and Kocher [27]. For 13 PCG genes, the values of nucleotide diversity (Pi), nonsynonymous substitution (Ka), synonymous substitution (Ks), and Ka/Ks were calculated using DnaSP v. 5 [28]. In these calculations, the mitogenome of *Chalcophora japonica* (Gory, 1840) was used as the reference.

### 2.3. Phylogenetic Analyses

A total of 35 species (Table 1) were used for phylogenetic analyses of Buprestidae, including four mitogenome sequences provided in the present study. Both maximum-likelihood (ML) and Bayesian inference (BI) trees were reconstructed using two data sets (PCGs and PCGs + rRNAs). The sequences of PCGs and rRNAs were aligned using MAFFT v. 7.313 and trimmed using trimAl v. 1.2 [29], which are integrated in PhyloSuite v. 1.2.2 [30]. The sequences of each species were concatenated using ‘concatenate sequence’ integrated in PhyloSuite. The best-fit models of the ML and BI methods were computed using ModelFinder [31]. The ML and BI trees were reconstructed using IQ-TREE v. 1.6.8 [32] and MrBayes v. 3.2.6 [33], respectively. The ML analyses were conducted with the following parameters: the number of bootstraps: 5000; replicates: 1000; and minimum correlation coefficient: 0.9. The BI analyses were performed with the following parameters: 5,000,000 generations, sampling every 100th generation, 2 independent runs, and a burn-in fraction of 0.25. In ML analysis, the nodal support was estimated through 2000 ultrafast bootstrap resampling. The Markov chain Monte Carlo chains were executed independently after excluding constant sites from the sequence alignment. The analyses were terminated when both runs achieved satisfactory convergence. A consensus tree was generated from the post-burn-in trees after discarding the first 25% of sampled trees from each run. The original trees were edited and visualized using Figtree v. 1.4.3.

**Table 1:** Information on the Buprestidae species and outgroup taxa used for phylogeny.

Taxa	Accession No.	Genome Size (bp)	A + T%	AT-Skew	References
*Agrilus sichuanus* Jendek, 2011	OK189519	16,521	71.73	0.12	[34]
*Agrilus adelphinus* Kerremans, 1895	NC071932	15,732	71.35	0.10	[35]
*Agrilus mali* Matsumura, 1924	MN894890	16,204	74.46	0.08	[36]
*Agrilus planipennis* Fairmaire, 1888	KT363854	15,942	71.90	0.12	[37]
*Agrilus discalis* Saunders, 1873	ON644870	15,784	74.59	0.11	[38]
*Agrilus zanthoxylumi* Li, 1989	OQ197496	16,320	74.70	0.08	[39]
*Coraebus diminutus* Gebhardt, 1928	OK189521	15,499	68.42	0.12	[34]
*Coraebus cloueti* Théry, 1895	OK189520	15,514	69.27	0.11	[34]
*Coraebus cavifrons* Descarpentries and Villier, 1967	MK913589	15,686	69.79	0.12	[40]
*Meliboeus sinae* Obenberger, 1935	OK189522	16,108	72.42	0.11	[34]
*Sambus femoralis* Kerremans, 1892	OK349489	15,367	73.23	0.12	[34]
*Sambus kanssuensis* Ganglbauer, 1890	OQ784265	15,411	72.4	0.10	[38]
*Habroloma* sp.	OQ784266	16,273	73.99	0.11	[38]
*Endelus continentalis* Obenberger, 1944	OL702762	16,246	75.60	0.13	[38]
*Cantonius szechuanensis* Obenberger, 1958	OQ784264	15,927	73.09	0.11	[38]
*Trachys auricollis* Saunder, 1873	MH638286	16,429	71.05	0.10	[41]
*Trachys troglodytiformis* Obenberger, 1918	KX087357	16,316	74.62	0.10	Unpublished
*Trachys variolaris* Saunders, 1873	MN178497	16,771	72.11	0.11	[42]
*Catoxantha luodiana* (Yang and Xie, 1993)	PP211020	15,594	68.68	0.12	[43]
*Anthaxia chinensis* Kerremans, 1989	MW929326	15,881	73.61	0.09	[44]
*Coomaniella dentata* Song, 2021	OL694144	16,179	76.59	0.01	[45]
*Coomaniella copipes* Jendek and Pham, 2013	OL694145	16,196	74.47	0.03	[45]
*Melanophila acuminata* (De Geer, 1774)	MW287594	15,853	75.66	0.02	[46]
*Nipponobuprestis guangxiensis* Peng, 1995	PP133641	15,775	65.33	0.14	[43]
*Chalcophora japonica* (Gory, 1840)	OP388437	15,759	67.97	0.13	[15]
*Chrysochroa fulgidissima* (Schönherr, 1817)	EU826485	15,592	69.92	0.15	[47]
*Dicerca corrugata* Fairmaire, 1902	OL753086	16,276	71.76	0.09	[45]
*Chrysochroa opulenta* (Gory, 1832)	PP211021	15,587	67.16	0.16	[43]
*Acmaeodera* sp.	FJ613420	16,217	68.41	0.11	[48]
*Ptosima chinensis* Marseul, 1867	OP388449	16,115	67.00	0.13	[15]
*Julodis variolaris* (Pallas, 1771)	OP390084	16,227	70.43	0.12	[15]
*Chrysobothris shirakii* Miwa and Chûjô, 1935	PV339621	15,789	78.54	0.02	This study
*Chrysobothris violacea* Kerremans, 1892	PV339622	15,961	79.29	0.02	This study
*Phaenops yin* Kubáň and Bílý, 2009	PV339623	16,051	76.51	0.04	This study
*Buprestisfairmairei* Théry, 1910	PV339624	13,390	73.42	0.09	This study
*Dryops ernesti* Gozis, 1886	KX035147	15,672	72.98	0.07	Unpublished
*Heterocerus parallelus* Gebler, 1830	KX087297	15,845	74.03	0.13	Unpublished

## 3. Results

### 3.1. Genome Organization and Base Composition

This study sequenced, assembled, and annotated the mitogenomes of *Chrysobothris shirakii* (GenBank No. PV339621), *Chrysobothris violacea* (No. PV339622), *Phaenops yin* (No. PV339623), and *Buprestis fairmairei* (No. PV339624). The average read coverage of *Chrysobothris shirakii*, *C. violacea*, *Phaenops yin,* and *Buprestis fairmairei* are 116.4 X, 82.2 X, 211.9 X, and 56 X, respectively. The number of paired-end reads is 15,220,144 (*C. shirakii*); 13,640,446 (*C. violacea*); 22,951,006 (*P. yin*); and 16,068,162, respectively. The lengths of these mitogenome sequences range from 15,789 bp to 16,051 bp, with the mitogenome of *Buprestis fairmairei* being 13,390 bp in size and encoding 35 genes, lacking the *trnV* and *12S* genes (Table 2). Except for the partial mitogenome of *Buprestis fairmairei*, the mitogenomes of the other three species are complete, circular, and double-stranded molecules. Each mitogenome contains 37 genes (13 PCGs, 22 tRNAs, and 2 rRNAs) and an A+T-rich region (control region, CR). Among the 37 genes, 14 (including *nad1*, *nad4L*, *nad4*, *nad5*, *trnQ*, *trnV*, *trnL1*, *trnP*, *trnH*, *trnF*, *trnY*, *trnC*, *rrnL*, and *rrnS*) genes are encoded on the N-strand, while the remaining 23 genes are encoded on the J-strand (Figures S1–S4).

These four mitogenome sequences all exhibit a high A + T bias, with *Chrysobothris shirakii* and *C. violacea* having an A + T content of 79.29%, higher than that of *Phaenops yin* (76.51%) and *Buprestis fairmairei* (73.42%). The AT-skew of these four mitogenomes is positive (0.02–0.09), while the GC-skew is negative (−0.2–−0.1). The gene intergenic spacers range from 1 to 30 bp in length, with the longest spacer located between the *trnC* and *trnY* of *Phaenops yin*. There are a total of 50 overlapping regions, with lengths ranging from 1 to 8 bp.

**Table 2:** The mitogenomes of *Chrysobothris shirakii*, *Chrysobothris violacea*, *Phaenops yin*, and *Buprestis fairmairei*.

Gene	Strand	Position From	To	Start Codons	Stop Condons	Intergenic Nucleotides
*trnI*	J	1/1/1/1	65/65/66/64			−3/−3/−3/−1
*trnQ*	N	63/63/64/64	131/131/132/132			−1/0/−1/−1
*trnM*	J	131/132/132/132	199/200/200/199			0/0/0/0
*nad2*	J	200/201/201/200	1225/1226/1223/1225	ATT/ATT/ATT/ATT	TAA/TAA/TAA/TAA	8/8/3/8
*trnW*	J	1234/1235/1227/1234	1301/1303/1296/1300			−8/−8/−8/−8
*trnC*	N	1294/1296/1289/1293	1357/1357/1356/1354			7/0/30/0
*trnY*	N	1365/1358/1387/1355	1429/1421/1452/1419			1/1/1/1
*cox1*	J	1431/1423/1454/1421	2961/2953/2987/2951	*/*/*	T(AA)/T(AA)/T(AA)/T(AA)	0/0/0/0
*trnL2*	J	2962/2954/2988/2952	3026/3018/3054/3016			0/0/0/0
*cox2*	J	3027/3019/3055/3017	3711/3703/3744/3698	ATA/ATA/ATA/ATA	T(AA)/T(AA)/TAA/T(AA)	0/0/4/0
*trnK*	J	3712/3704/3749/3699	3781/3774/3818/3768			0/-−/0/0
*trnD*	J	3782/3774/3819/3769	3847/3838/3886/3833			0/0/0/0
*atp8*	J	3848/3839/3887/3834	4003/3994/4048/3989	ATT/ATA/ATT/ATA	TAA/TAA/TAA/TAA	−7/−7/−7/−7
*atp6*	J	3997/3988/4042/3983	4671/4662/4716/4657	ATG/ATG/ATG/ATG	TAA/TAA/TAA/TAA	−1/−1/−1/−1
*cox3*	J	4671/4662/4716/4657	5454/5445/5504/5445	ATG/ATG/ATG/ATG	T(AA)/T(AA)/TAA/TAA	0/0/3/−1
*trnG*	J	5455/5446/5508/5445	5518/5508/5572/5508			0/0/0/0
*nad3*	J	5519/5509/5573/5509	5872/5862/5926/5862	ATT/ATT/ATT/ATT	TAG/TAG/TAA/TAG	−2/−2/2/−2
*trnA*	J	5871/5861/5929/5861	5933/5922/5993/5923			−1/−1/2/−1
*trnR*	J	5933/5922/5996/5923	5999/5988/6061/5988			−3/−3/−1/−1
*trnN*	J	5997/5986/6061/5988	6062/6051/6127/6051			0/0/0/0
*trnS1*	J	6063/6052/6128/6052	6130/6118/6194/6118			0/0/0/0
*trnE*	J	6131/6119/6195/6119	6196/6183/6259/6182			−2/−2/−2/−2
*trnF*	N	6195/6182/6258/6181	6258/6245/6322/6247			0/0/0/−1
*nad5*	N	6259/6246/6323/6247	7981/7968/8045/7965	ATT/ATT/ATT/ATA	T(AA)/T(AA)/T(AA)/TAA	0/0/0/0
*trnH*	N	7982/7969/8046/7966	8045/8033/8109/8029			0/0/0/0
*nad4*	N	8046/8034/8110/8030	9381/9369/9445/9365	ATG/ATG/ATG/ATG	T(AA)/T(AA)/T(AA)/T(AA)	−7/−7/−7/−7
*nad4L*	N	9375/9363/9439/9359	9665/9653/9729/9649	ATG/ATG/ATG/ATG	TAA/TAA/TAA/TAG	2/2/2/2
*trnT*	J	9668/9656/9732/9652	9733/9719/9795/9715			−1/−1/0/0
*trnP*	N	9733/9719/9796/9716	9797/9783/9861/9782			1/1/2/1
*nad6*	J	9799/9785/9864/9784	10,302/10,288/10,370/10,293	ATT/ATA/ATA/ATC	TAA/TAA/TAA/TAA	−1/−1/−1/1
*cytb*	J	10,302/10,288/10,370/10,293	11,441/11,427/11,512/11,435	ATG/ATG/ATG/ATG	TAG/TAA/TAG/TAG	−2/−2/−2/−2
*trnS2*	J	11,440/11,426/11,511/11,434	11,507/11,492/11,578/11,501			20/27/26/28
*nad1*	N	11,528/11,520/11,605/11,530	12,481/12,473/12,555/12,480	TTG/TTG/TTG/TTG	TAA/TAA/TAA/TAA	1/1/1/1
*trnL1*	N	12,483/12,475/12,557/12,482	12,547/12,539/12,621/12,545			0/0/0/0
*rrnL*	N	12,548/12,540/12,622/12,546	13,847/13,844/13,931/13,390			0/0/0/
*trnV*	N	13,848/13,845/13,932/*	13,917/13,914/14,001/*			0/0/0/
*rrnS*	N	13,918/13,915/14,002/*	14,652/14,699/14,780/*			0/0/0/
CR		14,653/14,700/14,781/*	15,798/15,961/16,051/*			

The order of four mitogenomes is *Chrysobothris shirakii*, *C. violacea*, *Phaenops yin*, and *Buprestis fairmairei*. * represents missing data.

### 3.2. Protein-Coding Genes, Codon Usage, and Nucleotide Diversity

The sequence lengths of the PCGs of *Chrysobothris shirakii* and *C. violacea* are both 11,159 bp, encoding 3710 amino acid residues; the sequence length of the PCGs in *Phaenops yin* is 11,171 bp, encoding 3715 amino acid residues; and the sequence length of *Buprestis fairmairei* is 11,163 bp, encoding 3710 amino acid residues. Among them, the PCGs length and amino acid residue count of *Phaenops yin* are slightly higher than those of the other three species.

**Figure 1:**
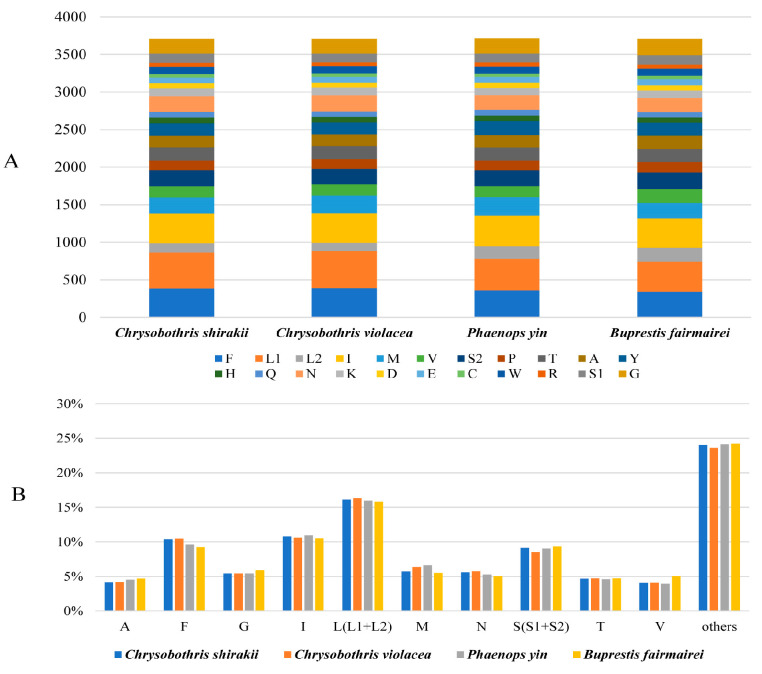
Numbers of different amino acids in the four new mitogenome sequences (**A**) and the percentages of the top eleven amino acids (**B**). The stop codons are not included in PCGs.

Except for the initiation codon of the *nad1* gene, which is TTG, the initiation codons of most PCGs are ATN. The initiation codon of the *cox1* gene could not be determined and may use a non-canonical initiation codon. For 13 PCGs, there are three types of termination codons: TAA, TAG, and T, with the single T as a termination codon being complemented by the 3′A residue on the mRNA. In these four mitogenome, L1, F, and I are three most frequently used amino acids (Figure 1).

**Figure 2:**
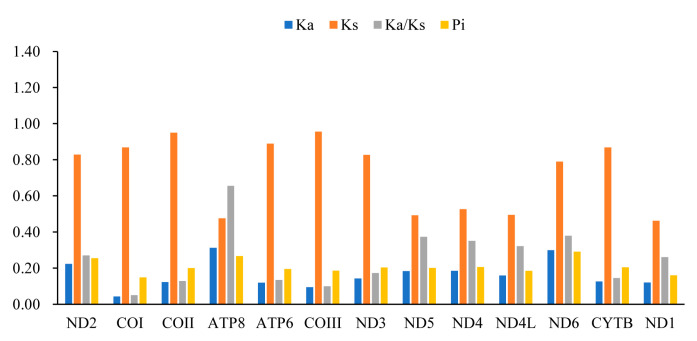
The Ka/Ks and Pi of PCGs in the four newly sequenced Buprestidinae mitogenomes. Pi: the values of nucleotide diversity; Ka: nonsynonymous substitution; and Ks: synonymous substitution.

A comparative analysis of the Ka/Ks ratios (Figure 2) of the four mitogenomes showed that the Ka/Ks value of the PCGs are all less than 1, indicating that these genes are under purifying selection. Among them, the ratio for *cox1* is the lowest (0.05). The Pi values of the PCGs in these four mitogenomes range from 0.149 (*cox1*) to 0.291 (*nd6*), with the highest nucleotide diversity level found in the *nd6* gene (Pi = 0.291), followed by *atp8* (Pi = 0.266).

**Figure 3:**
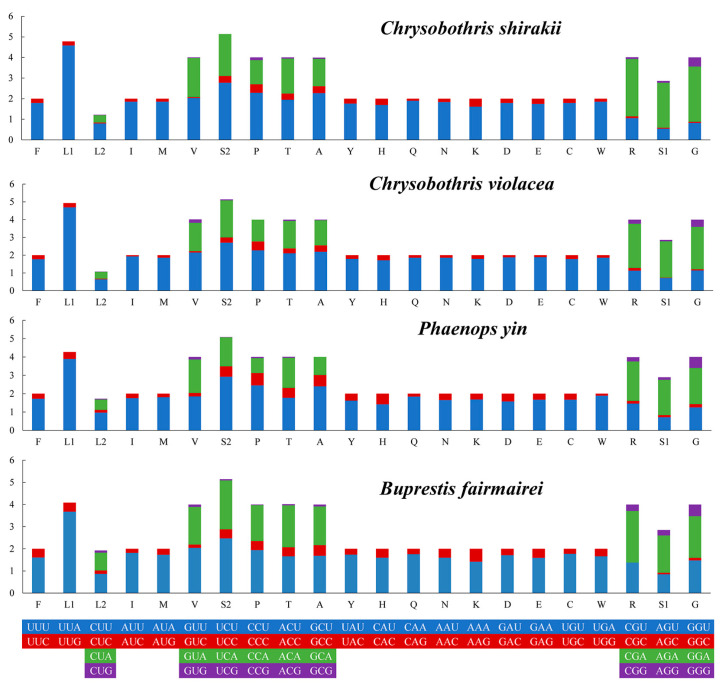
Relative synonymous codon usage (RSCU) of the four new mitogenome sequences. The codon families are displayed on the x-axis, with the codon usage frequency represented on the y-axis.

A comparative analysis of the codon usage frequencies (RSCU) for these four species showed that the most frequently used codons are UUU, UUA, and AUU (Figure 3).

### 3.3. Ribosomal and Transfer RNA Genes

Two rRNA genes are located between the CR region and *trnL1*, separated by *trnV*. The sequence length of the rRNA in the mitogenomes of the three species (*Chrysobothris shirakii*, *C. violacea*, and *Phaenops yin*) ranges from 2035 to 2090 bp, while the *12S* is missing in *Buprestis fairmairei*. The *16S* rRNA length in these four mitogenomes ranges from 845 to 1305 bp. The A + T content of the rRNAs in the three mitogenomes (*Chrysobothris shirakii*, *C. violacea*, and *Phaenops yin*) ranges from 79.85% (*Phaenops yin*) to 82.63% (*C. violacea*).

The tRNA sequence lengths in the four mitogenomes range from 1377 bp (*Chrysobothris violacea*) to 1457 bp (*C. shirakii*), with individual gene lengths varying from 62 bp (*trnA*) to 71 bp (*trnK*). Eight tRNA genes are located on the N strand, while the remaining 14 genes are located on the J strand. Among the 22 tRNA genes, except for *trnS1*, which lacks the DHU arm and cannot form a cloverleaf structure, the other 21 tRNA genes are able to form typical cloverleaf structures (Figures S5–S8).

### 3.4. Phylogenetic Relationships

**Figure 4:**
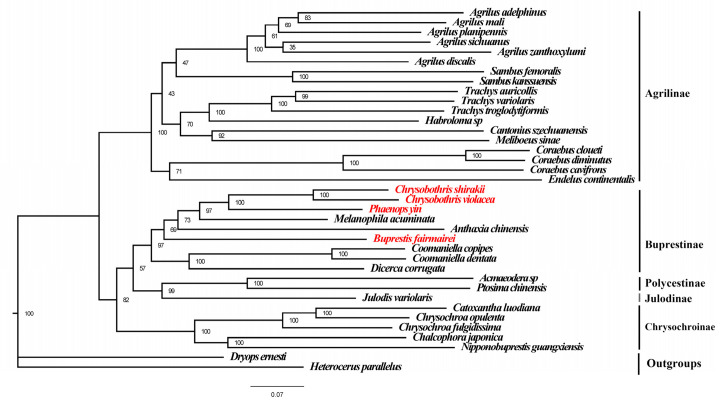
Maximum likelihood tree of Buprestidae based on PCGs + rRNAs. Values at nodes are ML bootstrap values. Red names represent the mitogenome data provided in this study.

**Figure 5:**
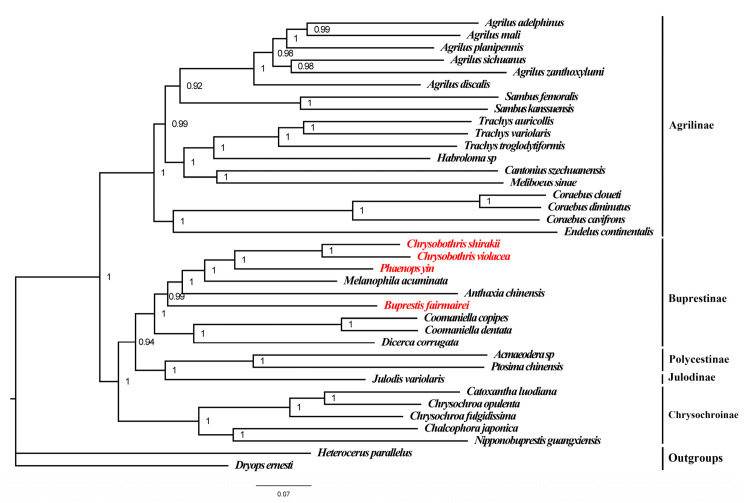
Bayesian tree of Buprestidae based on PCGs + rRNAs. Values at nodes are posterior probability. Red words are the target species provided in this study. Red names represent the mitogenome data provided in this study.

Based on the mitogenomes of the four species in this study and 32 previously known mitogenomes of Buprestidae, phylogenetic trees (ML and BI trees) of the Buprestidae family were constructed using the concatenated sequences of PCGs + RNAs. The results showed that the BI and ML trees have the same topological structure, with the phylogenetic relationships of the subfamilies as follows: (Chrysochroniae + ((Julodinae + Polycestinae) + Buprestinae) + Agrilinae) (Figure 4 and Figure 5). The four target species cluster together with other species of Buprestinae, with high support values (ML = 98, BI = 1). In Buprestinae, the phylogenetic relationships of the genus are as follows: ((*Dicerca* + *Coomaniella*) + (*Buprestis* + (*Anthaxia* + (*Melanophila* + (*Chrysobothris* + *Phaenops*)))). For the target genera, *Chrysobothris* is sister to *Phaenops*, and *Buprestis* is sister to (*Melanophila* + (*Chrysobothris* + *Phaenops*)) clade.

## 4. Discussion

Compared to the known species of Buprestidae [1], the number of reported mitogenomes is very limited. Currently, only 35 mitogenomes (including four mitogenomes provided in this study) have been reported [43]. In this study, the three complete mitogenomes contain 37 typical genes and a CR region, and the partial mitogenome of *Buprestis fairmairei*. Currently, the mitogenome composition of Buprestidae is consistent with that of other insects [10,11,12,13,49]. All four mitogenomes exhibit a high AT-skew. The gene order of these four mitogenomes is also consistent, aligning with the results from other species of the Buprestidae. In PCGs, the Ka/Ks of *cox1* is the lowest, suggesting that a large number of synonymous mutations have occurred in this gene, while *cox1* exhibits the highest level of conservation. Based on the most frequently used codons (UUU, UUA, and AUU), it can be inferred that the mitogenome exhibits a significant AT bias, which is consistent with previous studies [36,37,39–41]. Although gene rearrangements have been reported in some insects [50], this phenomenon has not been observed within Buprestidae to date.

Although different taxonomists have established various classification systems, research on the phylogeny of higher taxa within the Buprestidae is relatively scarce [9,51,52]. Firstly, the morphological characteristics were used to construct phylogenetic relationships among subfamilies [52]; however, the results of that study were severely inconsistent with the current classification system of Buprestidae, which is why it is seldom mentioned. Then, morphological characteristics were used to investigate the phylogenetic relationships among genera within Chrysochroina [53]. Subsequently, four gene fragments from 137 species [9] and their results supported the monophyly of Agrilinae, Julodinae, Galbellinae, and Polycestinae but did not support the monophyly of Chrysochroinae and Buprestinae. The relationships among tribes within Agrilinae are also unresolved. In recent years, mitogenome-based phylogenetic analysis has also been applied to Buprestidae [36,39,40,41,43], and the study results indicate that the phylogenetic relationships among subfamilies are relatively stable. The phylogenetic relationship of subfamilies is (Chrysochroniae + ((Julodinae + Polycestinae) + Buprestinae) + Agrilinae), which is largely consistent with mitogenome-based phylogenetic trees. In Buprestinae clade, *Coomaniella* is close to *Dicerca*, which is consistent with previous studies [9,45]. The phylogenetic tree of this study is consistent with recent research findings, possibly because both used mitochondrial genome data and the species used are largely the same. However, this result is inconsistent with the topology of the phylogenetic tree constructed based on gene fragments [9], which may be due to differences in the species and molecular data used in previous studies.

In this study, the mitogenomes of the genera *Buprestis*, *Chrysobothris*, and *Phaenops* are reported for the first time. The results indicate that *Chrysobothris* is close to *Phaenops*, and *Buprestis* is close to the clade composed of *Melanophila*, *Chrysobothris*, and *Phaenops*. Relying solely on morphological traits or molecular data to study phylogenetic issues is unreliable. These phylogenetic questions will be addressed based more mitogenome or genome data combined with morphological characteristics in the future studies.

## Data Availability

The complete mitogenome sequences are available in NCBI (PV339621, PV339622, PV339623, and PV339624).

## Supplementary Materials

The following are available online at https://www.mdpi.com/article/10.3390/genes16070828/s1, Figure S1. The mitogenome maps of Chrysobothris shirakii. Figure S2. The mitogenome maps of Chrysobothris violacea. Figure S3. The mitogenome maps of Phaenops yin. Figure S4. The mitogenome maps of Buprestis fairmairei. Figure S5. The secondary cloverleaf structure for the tRNA of Chrysobothris shirakii. Figure S6. The secondary cloverleaf structure for the tRNA of Chrysobothris violacea. Figure S7. The secondary cloverleaf structure for the tRNA of Phaenops yin. Figure S8. The secondary cloverleaf structure for the tRNA of Buprestis fairmairei.
