# Supplementary material for: Four Mitochondrial Genomes of Buprestinae (Coleoptera: Buprestidae) and Phylogenetic Analyses
Source: Genes (Basel). 2025 Jul 16;16(7):828. doi: 10.3390/genes16070828 (PMC12296024; doi:10.3390/genes16070828)
Supplement: Supplementary file 1 [file genes-16-00828-s001.zip › genes-3700039-Supplementary.pdf]

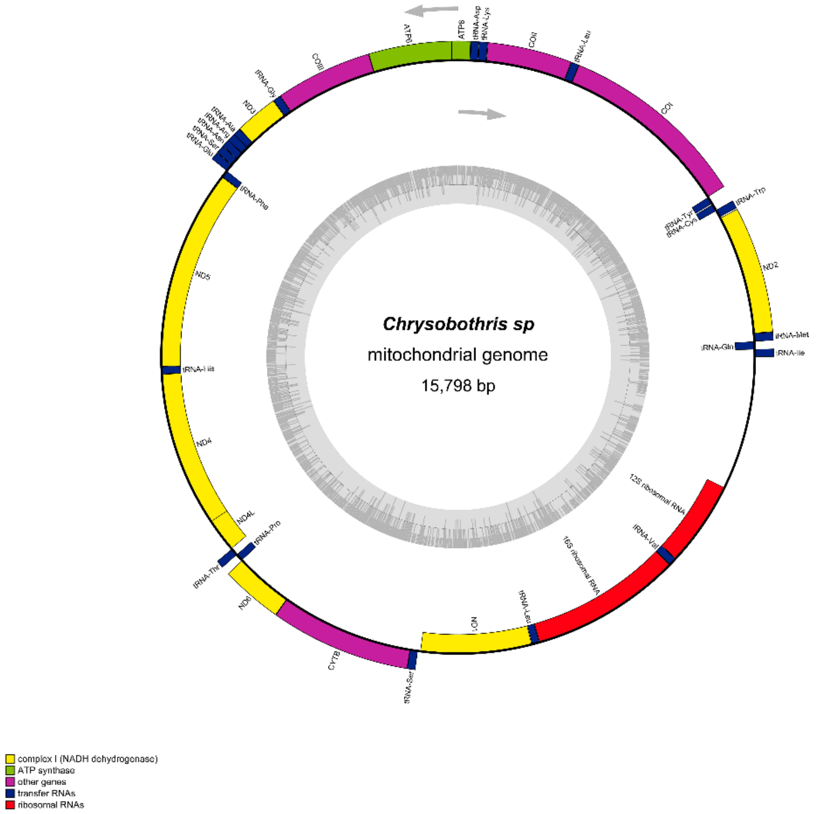

Figure S1. The mitogenome maps of *Chrysobothris shirakii*.

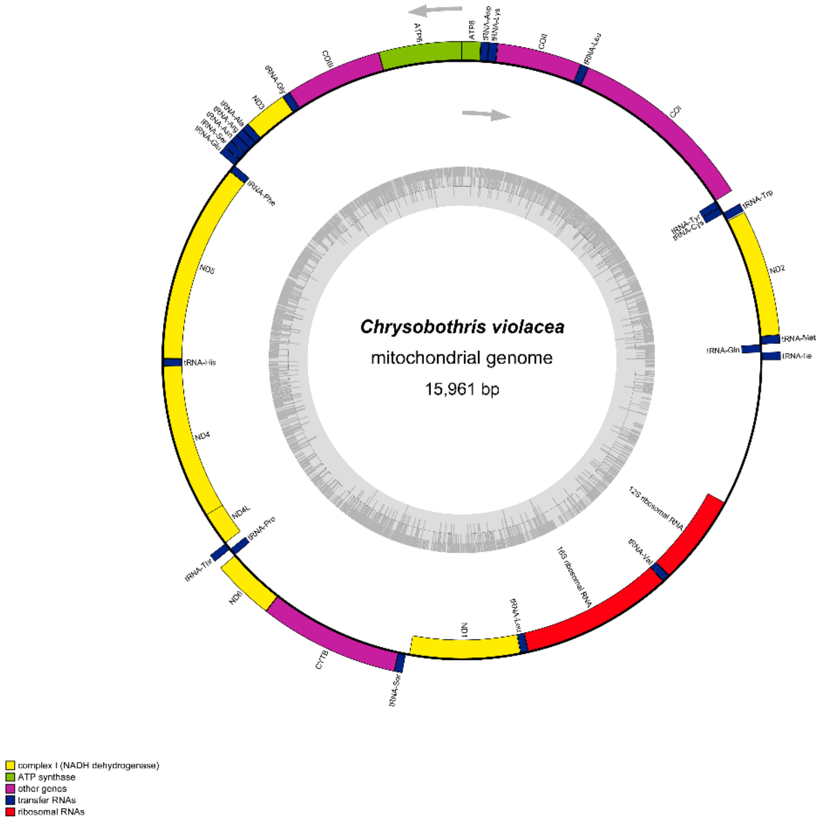

Figure S2. The mitogenome maps of *Chrysobothris violacea*.

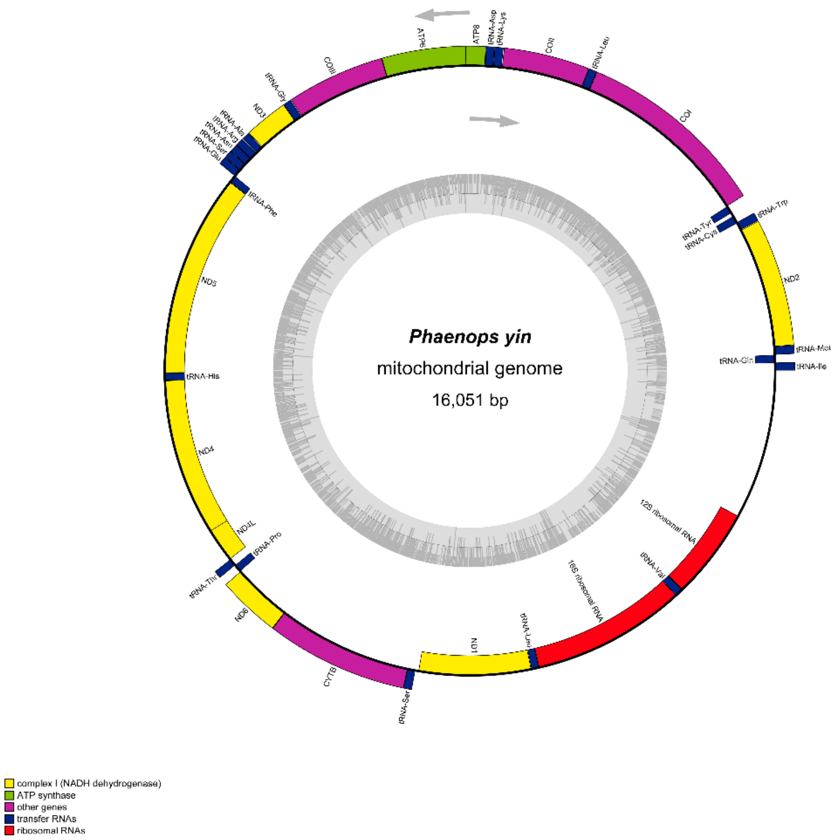

Figure S3. The mitogenome maps of *Phaenops yin*.

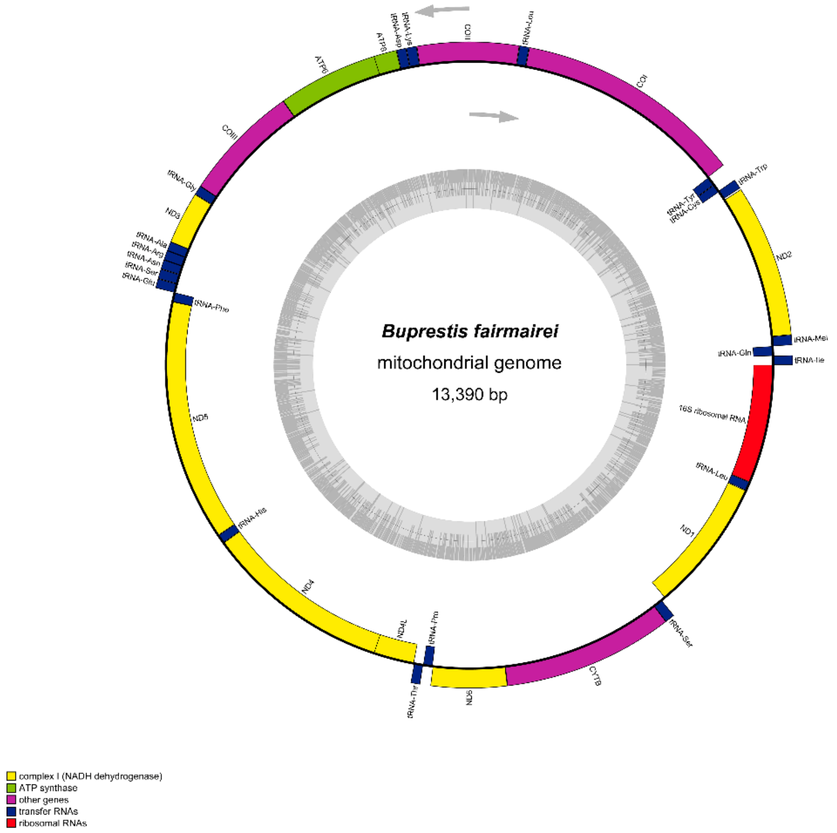

Figure S4. The mitogenome maps of *Buprestis fairmairei*.

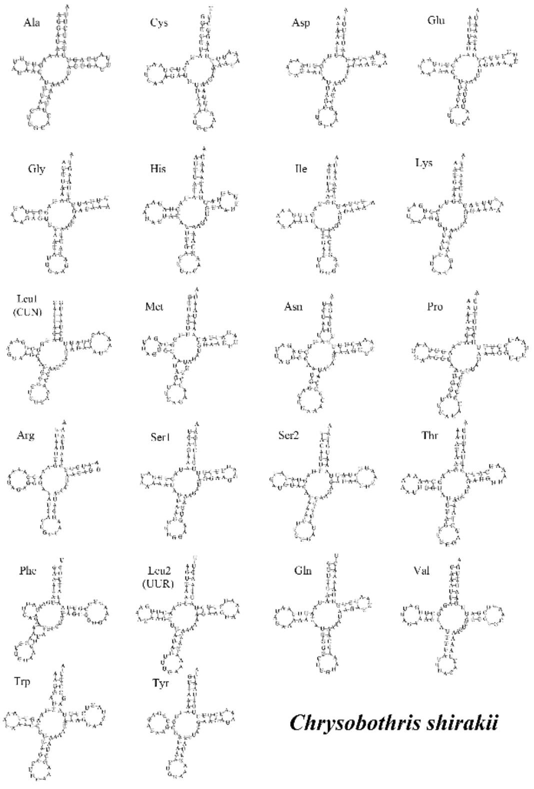

Figure S5. The secondary cloverleaf structure for the tRNAs of *Chrysobothris shirakii*.

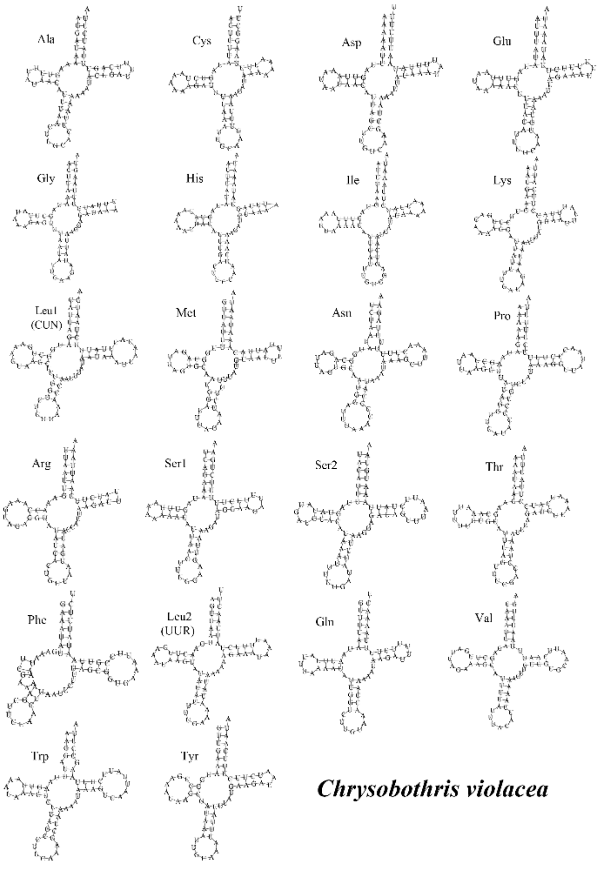

Figure S6. The secondary cloverleaf structure for the tRNAs of *Chrysobothris violacea*.

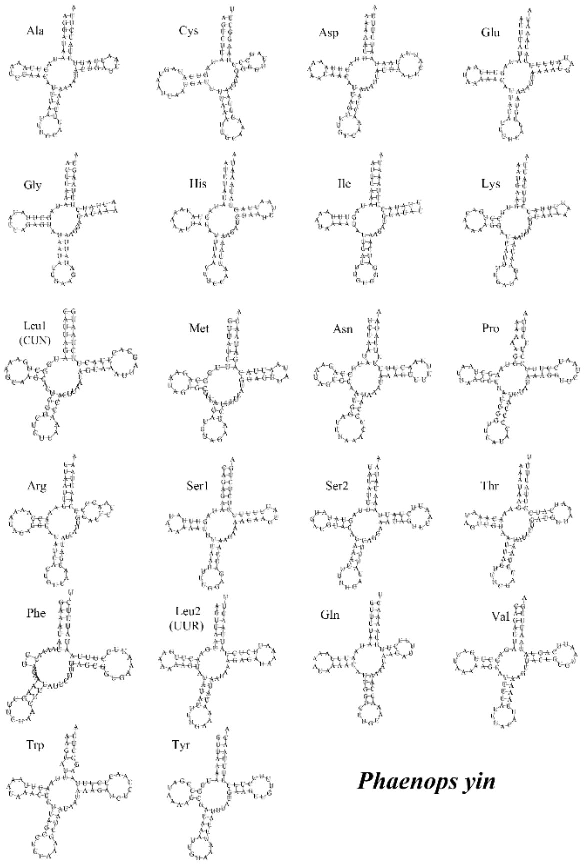

Figure S7. The secondary cloverleaf structure for the tRNAs of *Phaenops yin*.

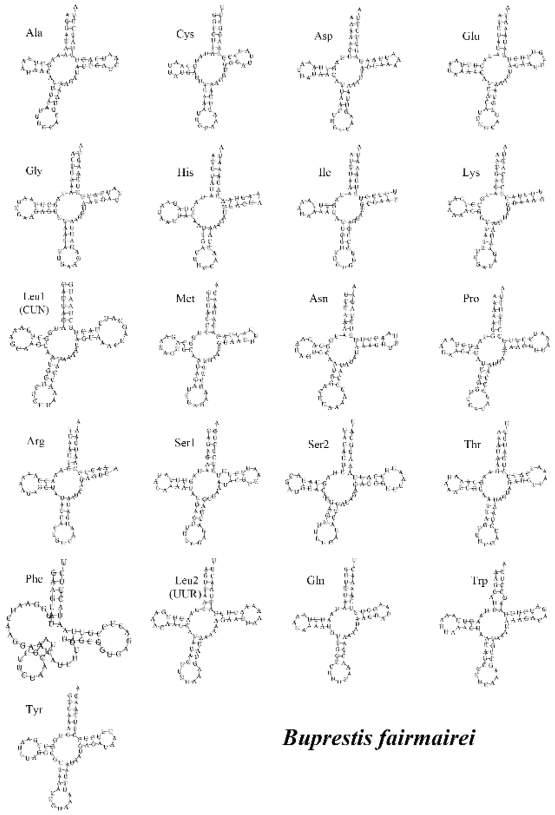

Figure S8. The secondary cloverleaf structure for the tRNAs of *Buprestis fairmairei*.
